# Kölliker's Organ and the Development of Spontaneous Activity in the Auditory System: Implications for Hearing Dysfunction

**DOI:** 10.1155/2014/367939

**Published:** 2014-08-20

**Authors:** M. W. Nishani Dayaratne, Srdjan M. Vlajkovic, Janusz Lipski, Peter R. Thorne

**Affiliations:** ^1^Department of Physiology, Faculty of Medical and Health Sciences, The University of Auckland, Private Bag 92019, Auckland 1142, New Zealand; ^2^Centre for Brain Research, Faculty of Medical and Health Sciences, The University of Auckland, Private Bag 92019, Auckland 1142, New Zealand; ^3^Section of Audiology, Faculty of Medical and Health Sciences, The University of Auckland, Private Bag 92019, Auckland 1142, New Zealand

## Abstract

Prior to the “onset of hearing,” developing cochlear inner hair cells (IHCs) and primary auditory neurons undergo experience-independent activity, which is thought to be important in retaining and refining neural connections in the absence of sound. One of the major hypotheses regarding the origin of such activity involves a group of columnar epithelial supporting cells forming Kölliker's organ, which is only present during this critical period of auditory development. There is strong evidence for a purinergic signalling mechanism underlying such activity. ATP released through connexin hemichannels may activate P2 purinergic receptors in both Kölliker's organ and the adjacent IHCs, leading to generation of electrical activity throughout the auditory system. However, recent work has suggested an alternative origin, by demonstrating the ability of IHCs to generate this spontaneous activity without activation by ATP. Regardless, developmental abnormalities of Kölliker's organ may lead to congenital hearing loss, considering that mutations in ion channels (hemichannels, gap junctions, and calcium channels) involved in Kölliker's organ activity share strong links with such types of deafness.

## 1. Introduction

As first described in 1863 by a Swiss anatomist and physiologist Albert von Kölliker, Kölliker's organ is an epithelial structure present in the developing auditory sensory organ in a wide variety of mammals, including cattle, rabbits, cats, dogs, and humans [1, 2]. It is one of the earliest visible epithelial structures of the developing cochlea and is the source of the sensory cells. After sensory cell differentiation, the residual Kölliker's organ remains as a large collection of epithelial cells on the medial aspect of the sensory organ, the organ of Corti (named after one of Kölliker's students, Alfonso Corti), while it is still in its developmental stage. As a transient structure, Kölliker's organ undergoes extensive remodelling in the embryonic or early postnatal stages and is eventually transformed into the inner sulcus region of the organ of Corti after the sensory structures become sensitive to external sound. Although Kölliker's organ was described well over a century ago, its function, especially in the period after sensory cell differentiation, is still largely unknown. This review looks at the structure and putative function of Kölliker's organ, with a major focus on purinergic intercellular signalling in the structure after sensory cell differentiation.

## 2. Morphology and Transformation

The cochlea develops from its base to apex in a time-dependent manner, both structurally and functionally. In the mouse, the putative sensory epithelium becomes visible around day 14 of gestation (embryonic day 14), when the endolymphatic duct is composed of tall columnar epithelial cells of ectodermal origin surrounded by mesenchymal tissue [3]. This mass of epithelial tissue which gives rise to the sensory IHCs is sometimes referred to as “Kölliker's organ” in the literature [4]. However, the sensory cell development and differentiation are not the topic of the review. Instead, it will focus on the functional role of Kölliker's organ after sensory hair cell differentiation and how genetic mutations may lead to abnormal function of this tissue and deafness.

As the sensory structures mature in the cochlea, the epithelium forms two domains starting from around embryonic day 16 (mouse): the greater epithelial ridge (GER) containing Kölliker's organ lying on its medial aspect and the lesser epithelial ridge (LER) in the lateral portion. The epithelial cells that separate these two regions become the inner and outer pillar cells of the organ of Corti [3]. While IHCs are thought to originate from the GER, the outer hair cells (OHCs) are derived from the LER [4]. The differentiation process between the sensory and nonsensory cells begins without any visibly distinguishable features between the two cell types, as nerve fibres begin to invade specific areas of the GER [5, 6]. Kölliker's organ in hamsters and other rodents initially appears in the basal turn, along with the lateral wall and Reissner's membrane, while the apical turn is still in its undifferentiated state [3, 7].

### 2.1. Structure of the Differentiated Kölliker's Organ

**Figure 1:**
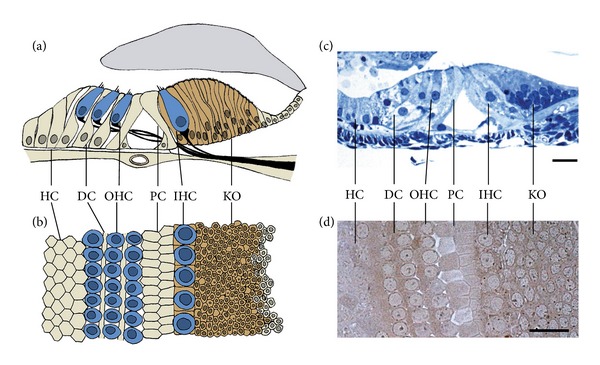
The immature organ of Corti and the adjacent Kölliker's organ. (a) A cross sectional diagram of a rat organ of Corti (approximately 10 days old) outlining the sensory hair cells and supporting epithelia. Kölliker's organ is found immediately adjacent to the inner hair cells (IHCs), shaded in darker colour (HC-Hensen's cells; DC-Deiters' cells; OHC-outer hair cells; PC-pillar cells; IHC-inner hair cells; and KO-Kölliker's organ). (b) A diagram outlining a horizontal section of the same developing organ of Corti, detailing the positions of various sensory and nonsensory cells. (c) Resin embedded toluidine-blue stained 1 *μ*m cross section of a 10-day-old Wistar rat organ of Corti. (d) Resin embedded horizontal section of an 11-day-old Wistar rat organ of Corti, postfixed in 1% osmium tetroxide. Scale bar: 20 *μ*m.

The differentiated Kölliker's organ is composed of tightly packed columnar epithelial cells, but due to its dense nature, the nuclei of these cells can be present in different regions of the cells (though mostly in the basal half), giving it a stratified appearance when viewed in cross section [8] (Figure 1). The supporting cells are generally separated by an extracellular space of about 200 Å, while some intercellular spaces measure as little as 30 Å [8–10]. In kittens, these supporting cells are approximately 65 *μ*m in height 3-4 *μ*m in width, and have microvilli covering their apical or luminal surface. An occasional kinocilium has been observed on the surface of the epithelial cells [8], although others have suggested that all cells within Kölliker's organ contain kinocilium surrounded by microvilli [10]. In mice, the microvilli are approximately 3-4 *μ*m in length and are present up to 10 days after birth [3]. The cytoplasm at the apical or luminal end of these cells is dense with organelles such as mitochondria, endoplasmic reticulum, and secretory vesicles [11]. Based on their morphological appearance, it was proposed that these cells secrete the tectorial membrane [12], as during early developmental stages (at birth in mice), the tectorial membrane is in contact with Kölliker's organ through a network of fine filaments, which later detach [3, 8, 10]. The tectorial membrane then extends to the OHC region two weeks after birth [3].

Cells of Kölliker's organ as well as other supporting cells in the immature organ of Corti are extensively connected through gap junctions, forming a syncytium [13]. Gap junctions are composed of two connexin hemichannels (connexons) from adjacent cells and are made of six subunits, each containing 4 transmembrane regions. These gap junctions are formed before the functional maturation of the cochlea [13] and comprise mainly connexin 26 (Cx26) and connexin 30 (Cx30) subunits (capable of forming heteromeric channels) which share similar expression patterns in Kölliker's organ epithelial cells in mice and are associated with congenital deafness [6].

### 2.2. Transformation

As the cochlea matures, columnar cells constituting Kölliker's organ are replaced by cuboidal cells, which are approximately 10 *μ*m in height, forming the mature inner sulcus [8]. The replacement process increases the intercellular space and greatly reduces the number of cells so that the final number of cuboidal cells is approximately 12% of the original columnar cell count [8]. This transformation occurs in a basal to apical and medial to lateral manner. Although the exact process behind this refinement is unclear, it may involve apoptosis of columnar cells, followed by formation of the inner sulcus containing cuboidal cells, while supporting cells lining the inner hair cells (border cells and phalangeal cells) are maintained through to the mature cochlea [8]. This transformation process appears to be very sensitive to thyroid hormone, as its deficiency leads to prolonged survival of Kölliker's organ in rats as old as 30 days and the malformed structure of the organ of Corti, particularly the tectorial membrane [11, 14, 15]. Supplementation of thyroid hormone at this age stimulates the transformation of the tall columnar cells of Kölliker's organ to the cuboidal cells of the inner sulcus [14].

## 3. Purinergic Signalling in Kölliker's Organ

Purines have been shown to have considerable influence on the cellular activity in Kölliker's organ. The actions of adenine compounds on cells and tissues were first described by Drury and Szent-Györgyi in 1929 [16]. However, the term “purinergic signalling” was not introduced until 1972, when adenosine triphosphate (ATP) was recognised as a neurotransmitter, followed by identification of purinergic receptors in 1976 [17]. Purines such as adenosine, ATP, and guanosine triphosphate (GTP) and pyrimidines such as uridine triphosphate (UTP) act as neurotransmitters, gliotransmitters, and paracrine signalling molecules in a variety of sensory systems including vision, smell, taste, and hearing [18]. In the peripheral auditory system, purines have multiple roles, including regulation of cochlear sensitivity and electrochemical homeostasis, synaptic transmission, and signalling between sensory and supporting cells after sensory cell injury [18]. There are two major classes of purinergic receptors, each with multiple subtypes. The P1 receptors (A_1_, A_2A_, A_2B_, and A_3_) are G protein-coupled and are activated by adenosine. These are further subdivided into receptors which stimulate the production of cyclic AMP (A_2A_ and A_2B_) and those which inhibit its production (A_1_ and A_3_). P2 receptors are classified into two major groups, P2X and P2Y (for review, see [19]). The P2X receptors are nonselective ATP-gated ion channels, with high permeability for Na^+^, K^+^, and Ca^2+^. P2X subtypes range from P2X1 to P2X7 with various roles throughout the body. On the other hand, P2Y receptors are G protein-coupled metabotropic receptors, which activate phospholipase C (PLC), resulting in activation of the second messengers diacylglycerol and inositol trisphosphate (IP_3_). IP_3_ increases intracellular Ca^2+^ levels by releasing these ions from internal stores [20]. P2Y receptors can be further subdivided into two groups. The first is mainly coupled to G_q_/G_11_, activating the PLC/IP_3_ pathway, and includes P2Y_1_, P2Y_2_, P2Y_4_, P2Y_6_, and P2Y_11_ [21]. The second group consists of P2Y_12_, P2Y_13_, and P2Y_14_ and is coupled to G_i/o_ and adenylyl cyclase [22].

### 3.1. P2 Receptors in the Developing Cochlea

In the developing rat cochlea, P2Y_1_, P2Y_2_, P2Y_4_, P2Y_6_, and P2Y_12_ receptors are expressed in sensory and nonsensory cells of the organ of Corti and the spiral ganglion neurons [23], although this review focuses on the cells of Kölliker's organ (for review of receptor expression in IHC and OHC, see [18]). P2Y_2_ and P2Y_4_ receptors are localised in cells of the GER [23] and are vital for its function. P2X_2-3_ and the transiently expressed P2X_3_ contribute to specific innervation of sensory cells by the spiral ganglion neurons (SGNs) [24]. During development, both type I and type II SGNs innervate IHCs and OHCs. This is followed in rodents by programmed withdrawal of the type I SGNs from the outer hair cells and the type II fibres from the inner hair cells few days after birth [24]. At this stage, SGNs are supported by neurotrophins secreted by the hair cells, such as brain-derived neurotrophic factor and neurotrophin 3 [25, 26]. P2X receptor signalling via P2X_2/3_ receptors inhibits this neurotrophic support [27]. P2X_7_ receptors are also expressed in hair cells and supporting cells within the cochlea from embryonic stages through to adulthood and are thought to be involved in ion homeostasis as well as apoptosis in the cochlea [28]. P2X_1_ receptors are transiently expressed during development and are downregulated by postnatal day 10 (P10) in rats, suggesting an involvement of this P2X subunit only in early stages of cochlear development [18, 29]. This may occur through regulation of cell death and differentiation [29].

### 3.2. Activity in Kölliker's Organ

Neural connections develop throughout the auditory system during development to form the necessary circuits. This formation and its refinement occur during a stage when there is no sound-driven activity (prior to the “onset of hearing”) in the cochlea. However, there is strong evidence for experience-independent action potentials throughout various regions of the developing auditory circuit, from the cochlear neurons to auditory nuclei in the brain [30, 31]. This is similar to other developing neural circuits, including the spinal cord [32], cerebellum [33], hippocampus [34], and the retina [35–39]. The origin of this neural activity was first found to be in the cochlea as tetrodotoxin applied to the round window membrane of the developing avian cochlea resulted in the elimination of neural activity throughout the developing auditory system [36]. The activity, therefore, appears to first begin spontaneously in the cochlea, generating action potentials in the auditory pathways through to the auditory cortex through the activation of IHC and their primary afferent neurons. The electrical activity in the immature auditory cortex thus appears to result from the auditory neural input in the absence of sound. Maintaining activity throughout the auditory circuit in this manner retains and refines the important synaptic connections made during very early development. Although spontaneous electrical activity was first observed in the developing IHCs over a decade ago [40], the link to refinement of the auditory system is only recently starting to emerge [31].

Tritsch et al. [35] identified Kölliker's organ as the area of the developing organ of Corti that could be responsible for generating the intrinsic spontaneous activity which drives the primary afferent auditory neurons [35]. Clusters (approximately 60 *μ*m wide) of supporting cells of Kölliker's organ show synchronous spontaneous activity, which could lead to a synchronous event involving adjacent IHCs because of their close proximity [35]. Their work on prehearing rats revealed spontaneous inward currents within epithelial supporting cells of Kölliker's organ and ruled out the direct involvement of IHCs or neural activity in initiating these events [30, 35, 41]; a finding that has been disputed by later studies [42–44] and is discussed in the next section. Furthermore, in the experiments by Tritsch and Bergles, spontaneous activity in supporting cells, IHCs, and SGNs was reduced by P2 purinergic receptor antagonists and extracellular ATP-hydrolysing enzymes (ectonucleotidases), suggesting an involvement of ATP released from Kölliker's organ and a subsequent activation of P2 receptors in surrounding cells. Predictably, experience-independent neural activity is transient, just like Kölliker's organ itself (it is only present during the prehearing stages of development). During the earliest stages of development (P0–3), the spontaneously generated currents are smaller, faster, and more frequent when compared to the activity observed later (P7–10) [30].

### 3.3. Possible Mechanisms of Spontaneous Activity Generation

As mentioned earlier, the possible involvement of purinergic signalling in generation of spontaneous activity in the auditory nerve was first introduced by Tritsch et al., as P2 receptor antagonists such as suramin and pyridoxal-phosphate-6-azophenyl-2′,4′-disulphonate (PPADS) inhibited experience-independent activity [35]. In addition, application of ATP to the epithelial nonsensory cells of Kölliker's organ resulted in bursts of excitatory postsynaptic currents in primary auditory afferents [30, 35]. Both P2X and P2Y receptors (in particular P2X_2_, P2X_7_, and P2Y_4_) are expressed in the developing organ of Corti, suggesting their likely involvement in ATP-induced currents [23, 41].

It is proposed that release of ATP from Kölliker's organ may occur through connexin hemichannels expressed extensively in Kölliker's organ [45, 46]. In particular, this could involve Cx26 and Cx30 which are expressed throughout the organ of Corti, excluding sensory cells [6]. This is supported by the inhibitory effects of gap junction blockers (such as octanol and carbenoxolone) on spontaneous activity as well as the observation of an increase of activity in response to hemichannel opening with Ca^2+^-free external solutions [30, 46, 47]. It is however important to note that pharmacological studies of such channels or hemichannels lack specificity, limiting their effectiveness in isolating the correct proteins [48]. Pannexins are another candidate for ATP release, as Panx1 and Panx2 are also strongly expressed in supporting cells of the adult cochlea [49], although their expression during development is not clear. Interestingly, genetic loci for the Panx1 gene also contain part of the gene sequence responsible for a dominant form of nonsyndromic sensorineural deafness (DFNA11) [50]. A cochlear organotypic culture study [47] provided further support for connexins as the channels responsible for ATP-induced Ca^2+^ waves over Panx1 and P2X_7_, though their work was limited to the outer sulcus [47]. While specific connexin activity has not yet been confirmed, it appears that ATP release in Kölliker's organ likely occurs through these hemichannels.

Therefore, as first proposed by Tritsch et al., the generation of intrinsic activity in the developing cochlea may include periodic release of ATP from inner supporting cells of Kölliker's organ, leading to P2 receptor activation at the adjacent IHCs, and subsequent glutamate release [35]. This, in turn, initiates bursts of action potentials in SGNs, connecting spontaneous activity of the cochlea to upstream neural activity in the auditory circuit. Studies carried out on epithelial cells of LER origin indicate that a large portion of Ca^2+^ rise following purinergic activation occurs from internal stores, as Ca^2+^ spikes persist in these cells even in Ca^2+^-free external solution [51–54]. Although developed from different domains, this may give an insight into mechanisms behind Ca^2+^ spikes observed during spontaneous activity generated in Kölliker's organ. While the rise in intracellular Ca^2+^ can lead to glutamate release from IHCs, it is also thought to be involved in inducing spontaneous morphological changes within Kölliker's organ. The ATP-induced activity is likely biphasic, as the P2X receptors respond more rapidly than P2Y receptors [35]. This is evident during the response of supporting cells to the specific P2Y receptor agonist UTP, which is consistently slower than the responses elicited by ATP [35]. Following its release from supporting cells, ATP is likely degraded through the actions of ectonucleotidases (extracellular ATP-hydrolysing enzymes), resulting in generation of adenosine [55]. Although adenosine stimulates P1 (adenosine) receptors in the adult cochlea, it does not have any effect on the spontaneous activity in the developing organ of Corti [35]. The combined action of ATP release through connexins and its continuous degradation in the extracellular space by ectonucleotidases may be responsible for the rhythmic regulation of spontaneous activity. A recent study on adult guinea pigs also suggested the involvement of ATP in mediating gap junctional coupling [56]. This study demonstrated an uncoupling effect on gap junctions by ATP in cochlear supporting cells, an effect mediated by P2X rather than P2Y receptors [56].

In contrast to the purinergic mechanism proposed by Tritsch et al. [35], more recent findings suggest that the initiation of spontaneous activity occurs through IHCs, without ATP-induced depolarisation [42–44]. Johnson et al. [43] recorded sustained spontaneous activity from mouse IHCs which was independent from purinergic signalling by Kölliker's organ. In addition, IHCs still fire spontaneous action potentials in the presence of broadly selective P2 receptor antagonists such as PPADS and suramin and in the absence of the P2X_4_ receptor subunit [43, 44]. Both studies revealed a modulatory role of the efferent neurotransmitter acetylcholine using *α*9*α*10 nicotinic receptor antagonist strychnine [43, 44]. This again challenges the original work by Tritsch et al. [35], which reported no change of spontaneous activity in response to strychnine [35]. The major differences between the purinergic and IHC theory of spontaneous activity generation may be due to variation in ionic compositions (such as K^+^ concentrations and Ca^2+^ buffering), which may affect the resting potential of IHCs and therefore their ability to generate action potentials. However, if ATP release by Kölliker's organ is not responsible for generating spontaneous activity which drives the auditory system prior to the “onset of hearing,” questions remain regarding the role of spontaneous purinergic activity in Kölliker's organ.

### 3.4. Rhythmic Morphological Changes within Kölliker's Organ

Strongly correlated with the onset of inward currents, supporting cells of Kölliker's organ undergo spontaneous morphological changes where the cytoplasm pulls away from the membrane, resulting in crenation of the cell and increased extracellular space between individual cells. These events are tightly linked as demonstrated by the fact that 93% of optical changes resulting from cell shrinking are correlated with inward currents [35]. This allows real-time visual detection of the spontaneous activity in Kölliker's organ by recording changes in the optical density/refractive indices of supporting cells involved. Such optical changes occur at a frequency of 0.034 ± 0.003 Hz and could be observed at random locations within the length of Kölliker's organ [32]. Morphological changes are relatively specific to Kölliker's organ but are also observed in the processes of phalangeal cells away from the OHCs [41]. Much like the inward currents, these spontaneously generated morphological changes are also inhibited by nonselective P2 receptor antagonists such as suramin and activated by both ATP and UTP, suggesting a similar purinergic control [30, 35, 41]. Although spontaneous currents and Ca^2+^ waves are present in Kölliker's organ from birth in rodents, morphological activity is only observed a few days after birth, indicating that a certain level of development is required to induce changes in cell shape. While low expression of purinergic receptors is a possibility, ATP still induces inward currents and Ca^2+^ waves in Kölliker's organ of young rodents, suggesting that the expression levels of purinergic receptors cannot solely account for the lack of morphological changes at these earlier stages [23, 35]. In order to identify the mechanisms of the morphological changes, supporting cells of Kölliker's organ were depolarised in the presence of high intracellular Ca^2+^ concentrations. Neither large current injections nor rise in extracellular K^+^ resulted in the spontaneous morphological changes [41]. In contrast, rises in intracellular Ca^2+^ alone induced changes in cell diameter of the activated cell as well as adjacent cells. These results indicate that intracellular Ca^2+^ rise alone can lead to rhythmic morphological changes, which spread to adjacent cells within Kölliker's organ [41].

A possible mechanism for the changes in cell shape involves the activation of Cl^−^ channels or nonselective cation channels by Ca^2+^, leading to the expulsion of water. It has been suggested that cultured cochlea has the ability to secrete water, providing further support to this theory [57]. Other possibilities include the involvement of contractile proteins such as actin, although there is a lack of evidence to support this. If these morphological changes in the supporting cells do in fact result in the secretion of water, this process may be involved in forming the cochlear compartmental fluids (endolymph and perilymph) during development.

### 3.5. Calcium Signalling in Kölliker's Organ

Calcium is a major intracellular messenger in the cochlea, being involved in a number of signalling pathways. Within Kölliker's organ, a strong correlation was initially observed between spontaneously generated currents, morphological changes, and Ca^2+^ spikes. This likely occurs through the combined rise in intracellular [Ca^2+^] from internal stores and inward currents through P2X receptors-channel complexes [30, 35]. The Ca^2+^ waves in Kölliker's organ are comparable to those found between groups of astrocytes connected via gap junctions [58]. Each wave is initiated in a small group of cells (one to four) and then spreads radially. Ca^2+^ waves are also observed within the outer sulcus cells following ATP release or mechanical stimulation [51]. These Ca^2+^ waves lead to further ATP release, resulting in a regenerative wave that allows the synchronisation of nearby cells [30, 35]. ATP-induced Ca^2+^ waves can be observed from very early stages of development, with their frequency increasing dramatically (by 5-fold), along with an increase (1.7-fold) of the area of activation [30]. Although extracellular ATP can induce Ca^2+^ waves throughout Kölliker's organ at very early stages (P0-P1), naturally occurring Ca^2+^ waves are rare at that age, possibly due to lower levels of ATP release. The ATP-induced currents are also of smaller amplitudes at that age [30, 41].

Clinically, lack of regular Ca^2+^ action potentials in IHCs during development could lead to hearing impairment, particularly due to defects in the Ca^2+^ channel Ca_v_1.3, as its dysfunction is detrimental to cochlear functioning [59]. This particular *L*-type channel is the predominant Ca^2+^ channel in IHCs of developing cochlea and is responsible for exocytosis of glutamate and potentially of other neurotrophic factors [60]. It is likely that Ca^2+^ is also involved in rhythmic morphological changes within supporting cells of Kölliker's organ. One intriguing possibility is that the increase in intracellular Ca^2+^ within supporting cells can activate Ca^2+^-activated Cl^−^ channels, leading to efflux of Cl^−^. A recent study has demonstrated a strong expression of Ca^2+^-activated Cl^−^ channels (Anoctamin-1) in Kölliker's organ, particularly in supporting cells immediately adjacent to IHCs [61]. While Anoctamin-1 is thought to be involved in pacemaker activity and fluid secretion in the digestive system [62], its functional significance in Kölliker's organ is yet to be established. The osmotic gradient created by this Cl^−^ efflux could in turn cause the movement of water out of these cells, resulting in morphological changes within Kölliker's organ [35, 41]. This is supported by the negative effect of Cl^−^ channel inhibitor 4,4′diisothiocyano-2,2′stilbene disulfonic acid on spontaneous morphological activity in Kölliker's organ [41].

## 4. Kölliker's Organ and Deafness

The role of Kölliker's organ is quickly evolving from simply providing support for adjacent IHCs, to potentially initiating activity that is necessary for the development of a fully functional auditory system. While no direct link between Kölliker's organ dysfunction and deafness has yet been established, critical components of its organisation appear to be linked to major forms of deafness. One of the strongest potential links lies within mutations of the* GJB2* gene, which encodes Cx26 and accounts for about 50% of prelingual childhood deafness [63]. Both Cx26 and Cx30 (with strong links to both syndromic and nonsyndromic congenital hearing loss [64, 65]) are highly expressed throughout Kölliker's organ and are likely to play a key role in generating and transmitting synchronised spontaneous activity. In rodents, mutations and blockers of Cx30 reduce Ca^2+^ transients within Kölliker's organ, and the Cx30-null mice also show an elevation of auditory thresholds [66, 67]. Furthermore, Ca_v_1.3 channels are essential in creating and maintaining synapses during the critical spontaneous activity driven period of development, and adverse effects are seen as early as the first postnatal week in their absence, starting with OHC degeneration [59].

In addition, the function as well as the transformation of Kölliker's organ to the inner sulcus is crucial for the maturation of the cochlear structure and function. Thyroid gland deficiencies have been shown to affect this process by maintaining Kölliker's organ past the onset of hearing, and in humans, impairment of thyroid hormone signalling is associated with hearing loss [68–70]. This may be due to malformed structural changes during development, although its exact cause and incidence are still not well characterised (for review, see [71]).

## 5. Summary

First discovered over a century ago, Kölliker's organ is present transiently in the developing cochlea, until the cochlea becomes sensitive to external sound. It is an epithelial structure composed of long columnar cells and lies immediately adjacent to IHCs. From this close proximity, epithelial cells of Kölliker's organ have been suggested to initiate spontaneous electrical activity in the IHCs, driving action potentials in the primary auditory neurons, and auditory nuclei in the brainstem. Although the exact mechanism remains unclear, rhythmic ATP release from connexin hemichannels and activation of purinergic P2 receptors may contribute to rhythmic current/morphological oscillations of Kölliker's organ. The rise in intracellular Ca^2+^ or ectonucleotidase activity may act as a feedback for ATP release and thus control the rhythmicity of ATP release in the developing cochlea. Interestingly, both types of ion channels (connexins and P2 receptors) have been linked to hearing loss. Following sound detection by the cochlea (the “onset of hearing”), Kölliker's organ disappears, transforming into the adult inner sulcus region which contains cuboidal epithelial cells. From a physiological perspective, this is an important step in cochlear adaptation to external environment. From a developmental point of view, purinergic signalling in Kölliker's organ may provide a sophisticated mechanism central to the tonotopic organisation of the cochlea which starts to develop in the absence of sound.

## Abbreviations

ATP: Adenosine triphosphate
Cx: Connexin
GER: Greater epithelial ridge
IHCs: Inner hair cells
IP_3_: Inositol trisphosphate
LER: Lesser epithelial ridge
OHCs: Outer hair cells
PLC: Phospholipase C
PPADS: Pyridoxal-phosphate-6-azophenyl-2′,4′-disulphonate
SGNs: Spiral ganglion neurons
UTP: Uridine triphosphate.
